# Global research trends in CRISPR-related technologies associated with extracellular vesicles from 2015 to 2022: a bibliometric, dynamic, and visualized study

**DOI:** 10.1186/s11658-023-00507-z

**Published:** 2023-12-02

**Authors:** Jianjing Lin, Shicheng Jia, Zilu Jiao, Jiayou Chen, Wei Li, Fuyang Cao, Xintao Zhang

**Affiliations:** 1 Department of Sports Medicine and Rehabilitation, Peking University Shenzhen Hospital, Shenzhen, Guangdong China; 2 Shantou University Medical College, Shantou, Guangdong China; 3 Department of Orthopedics, Second Hospital of Shanxi Medical University, Taiyuan, Shanxi China

**Keywords:** CRISPR-related technology, Extracellular vesicles, Bibliometric, Visualization

## Abstract

**Purpose:**

This study aims to explore the emerging trends, dynamic development, and research hotspots of clustered regularly interspaced short palindromic repeats (CRISPR) technology associated with extracellular vesicles during the past 7 years and demonstrate them by visualization.

**Methods:**

A total of 219 records related to CRISPR technology associated with extracellular vesicles from 2015 to 2022 in the Web of Science Core Collection (WoSCC) database were collected. R language, VOSviewer, CiteSpace, and GraphpadPrism software packages were used to analyze the history of this research, the general characteristics of the literature, and keywords. Finally, the hotspots and latest trends in CRISPR technology associated with extracellular vesicles are predicted.

**Results:**

A total of 219 articles were collected for this study. The production of publications about CRISPR technology associated with extracellular vesicles has increased annually. Researchers from China, the USA, and Germany made the most important contributions to this trend, while RLUK Research Libraries UK offers the largest amount of literature in this field. Shenzhen University, Nanjing Medicine University, and Peking University exhibited the closest cooperation. Additionally, active topics burst during different periods, as identified according to 317 keywords belonging to 39 disciplines. Keywords were clustered into seven research subareas, namely exosome, nanovesicles, DNA, gene editing, gene therapy, cancer therapy, and endometrial stromal cells. The alluvial map of keywords reveals that the most enduring concepts are gene therapy, nanovesicles, etc., while the emerging keywords are genome, protein delivery, plasma, etc.

**Conclusions:**

We reviewed 219 previous publications and conducted the first bibliometric study of CRISPR technology related to extracellular vesicles from 2015 to 2022. This comprehensive summary constructed a knowledge map and demonstrates the trends in this area. The current trends and potential hotpots for this topic are also identified, which will be a great help for researchers in the future.

## Introduction

Regarding the exploration of gene therapy and biological mechanisms, modulation and edition of hereditary information are key steps. Benefiting from the development of genome engineering (GE), clustered regularly interspaced short palindromic repeat (CRISPR)/CRISPR-associated (Cas) has been regarded as a revolutionary technology for gene editing and transcription modulating since 2012 [1, 2]. Moreover, compared with zinc finger nucleases, recombinases, meganucleases, transcription activator-like effector nucleases, and restriction enzymes, although it enjoys unparalleled advantages such as precise editing of multiple target sites, rapid generation of mutants, and single-guide RNA (sgRNA) designability [3], its components should be delivered via special tools under strict conditions. An ideal delivery system for CRISPR components should be highly safe, stable, efficient, and nontoxic [4]. Conventional viral carriers are limited by carcinogenesis, packaging constraints, produced efficiency, immunogenicity, and the lifetime of Cas expression [4]. Nonviral carriers should address issues such as rapid clearance, biocompatibility, toxicity, and release of active ingredients [4, 5].

Recently, this approach has seen increased popularity in the field of delivering genetic components through extracellular vesicles (EVs) [4]. EVs are functional materials secreted by various natural cells under different external or internal conditions. The verified components within them can regulate biological processes through signal pathways [6, 7]. EVs exhibit high biocompatibility and stability due to their phospholipid bilayer membranes and high-level signal molecules on their surface [6–8]. Additionally, they show good modifiability and reduced immune reactions [9, 10]. They can also achieve specific and targeted delivery by surficial ligands and anchor sites [11]. Significantly, substances encapsulated within them have lower clearance rates and improved circulation longevity [6–8]. Therefore, either artificially modified or natural EVs are reliable, and promising for delivery of CRISPR-related components with high safety. However, owing to various disturbances, the exact delivery of components via EVs suffers from issues. Despite numerous reviews that have summarized various projects based on CRISPR/Cas systems from different aspects and strategies for EV-based delivery systems of CRISPR-related components with corresponding details [4, 12, 13], the meaningful and comprehensive analysis of such publications in the field remains significantly insufficient and requires an urgent summary. Besides, it is necessary to predict the current hot topics and potential directions in this field in a visual and metrological view.

Publications are regarded as a valuable indicator of research trends, core research results, and meaningful contributions. Thus, bibliometric analysis has been widely conducted to summarize and identify numerous characteristics of papers and demonstrate the corresponding trends [14]. Such study can reveal the evolution of publications, evaluate advanced issues, and predict research hotspots in related fields through citation networks [15–17]. Although software and multiple codes for statistics can be used to analyze and relate publications through visualization [18–20], we have not found enough published bibliometric studies on CRISPR-related technologies associated with EVs, and similar analytical reports on the use of EVs in CRISPR-related technologies are lacking. Therefore, we conducted this bibliometric study to supplement related knowledge. This paper presents a study of the literature on CRISPR-related technologies associated with EVs and a visualized analysis of such publications from 2015 to 2022, to explore the emerging trends, dynamic development, and research hotspots.

## Methods

### Data source and search strategy

The Web of Science Core Collection (WoSCC) database is derived from Clarivate Analytics and contains more than 12,000 international academic journals, being recognized as a comprehensive and authoritative database [21]. Therefore, on the basis of previous studies, we used WoSCC to acquire worldwide information for bibliometric analysis [22–24]. All published literature was obtained from WOS, searched from 2015 to 2022, and retrieved on 16 May 2023. In this study, the search strategy was as follows: Theme = Crispr AND Theme = (Extracellular Vesicles or Exosomes or Ectosomes or Apoptotic bodies or Microvesicles or Oncosomes or Microparticles) AND Published year = (2015.01.01–2022.12.31) AND Document type = (article or review) and Language = (English). We included publications according to (1) manuscripts in the field of CRIPSR-related technology and EVs, (2) publication type of Review or Article, and (3) English language. We excluded publications according to (1) publications not associated with the search strategy, and (2) publications that were meeting abstracts, news, briefings, etc.

### Data collection and statistics

We refined the details of countries and regions in WoSCC by indexing the countries/regions searched. All selected data for the publications such as authors, title, national origin, year of publication, alliance, abstract, keywords, and names of journals were stored in downloadable format. J.J.L. and S.C.J. independently set, searched, and filtrated all the data collected. All dissenting opinions were subjected to multiple discussions within the group and repeated consultations with professionals to draw the final conclusions. All collaborators cleaned, analyzed, and summarized the data using GraphPad Prism 8 and Origin 2021.

### Bibliometric analysis

The number of publications and their citations can be directly described by WoSCC, which we use to summarize the relative research interest (RRI). RRI is used to reflect the ratio of all publications in the research area of interest each year to the total number of publications in that year [25, 26]. The publishing years were set from 2015 to 2022. All these data were analyzed by using GraphPad Prism 8. We used R software, including scipy, matplotlib, numpy, and python, to obtain a world map. WoS and GraphPad Prism 8 were used to access and analyze the total publications from top countries. Furthermore, The *H*-index describes a scholar who has published *H* papers and has been cited at least *H* times, which is regarded as a measure of the impact of their research [27]. We analyzed the *H*-index by using GraphPad Prism 8. Analysis of authors, institutions, highly cited journals, and research orientations was also performed by using corresponding software.

### Visualization

We used the VOS viewer software to build the network of literature in this project. Analysis of co-citation, co-occurrence, and coupling relationship of bibliography were also conducted using this software. Additionally, R language was used to enable the visualization of publications and their corresponding relationships between countries. Finally, we used CiteSpace (6.1.R2) to summarize related high-quality journals with citation bursts, to identify strong citation bursts of keywords and references and to conduct a cluster analysis of co-citation of keywords.

## Results

### Overview of global literature

**Fig. 1:**
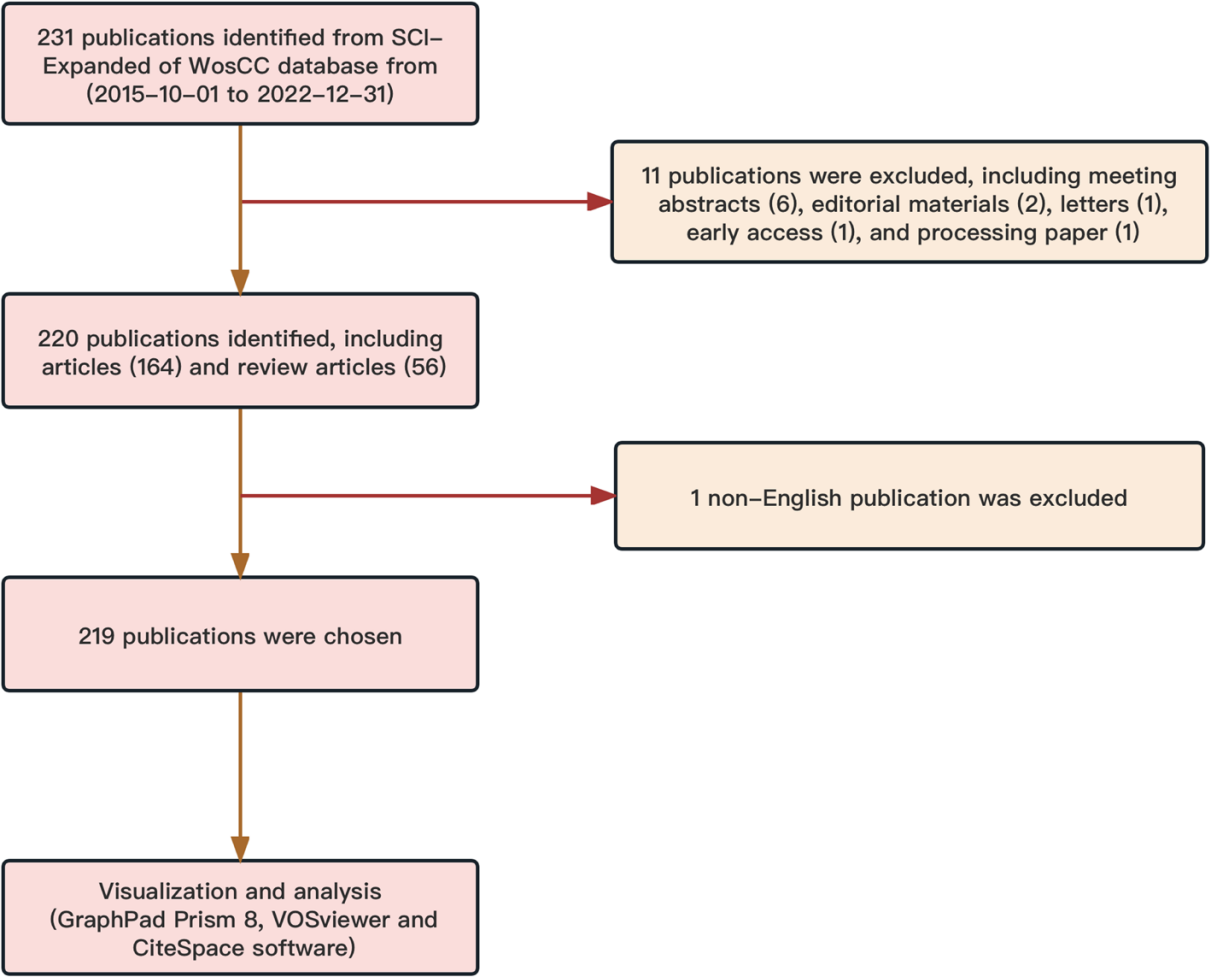
Flowchart of the screening of the retrieved publications for this bibliometric analysis. Purple blocks represent the remaining sections after each cull, while orange blocks represent the culled literature. Numbers in parentheses are the corresponding number of publications. A total of 231 relevant publications were collected from 2015 to 2022 After excluding 6 conference abstracts, 2 editorial materials, 1 letter, 1 early-access publication, and 1 processing paper, a total of 220 publications were retrieved (including 164 articles and 56 reviews). Excluding 1 not in English language, 219 publications were finally identified

**Fig. 2:**
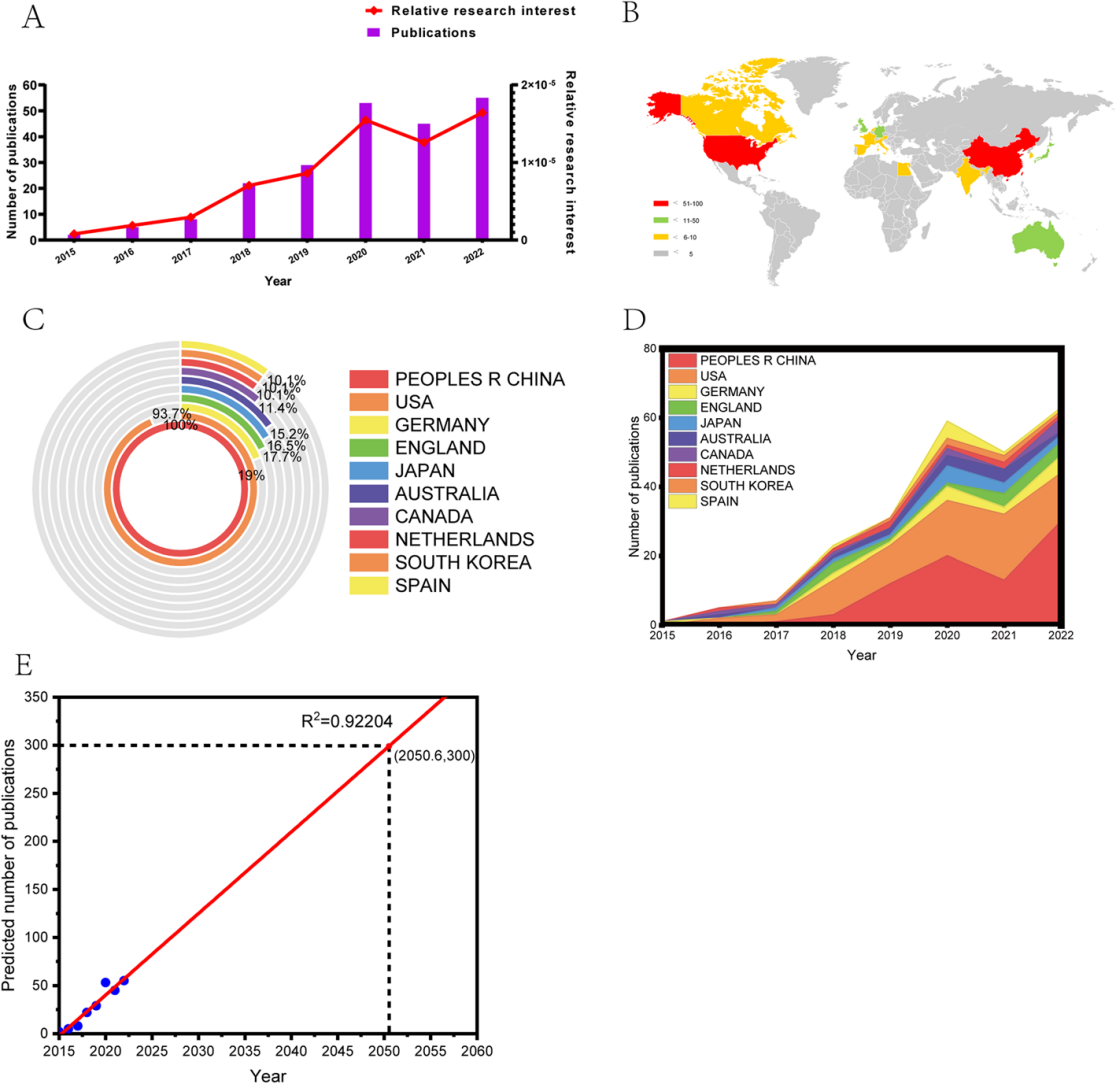
General trend of related publication worldwide from 2015 to 2022. **A** The trend of RRI and number of publications over time. **B** The distribution of publications among countries. **C** The top 10 countries in the field and the proportion of different countries relative to China. **D** An alluvium plot of the number of publications in the top 10 countries over time. The area size represents the number of publications, while the slope of the line segment represents the growth rate of publications. **E** A linear regression plot based on curve fitting of the global publication volume from 2015 to 2022, predicting that there will be a total of 300 articles published by the middle of 2050

Using the strategy described above, a total of 231 relevant publications were collected from 2015 to 2022 After excluding six conference abstracts, two editorial materials, one letter, one early access publication, and one processing paper, a total of 220 publications were retrieved (including 164 articles and 56 reviews). Excluding 1 not in English language, 219 publications were finally identified (Fig. 1). Figure 2A shows that the trend of volume in the global papers fluctuates but has generally increased year on year, especially since 2019. The annual number of published papers has basically remained above 30, indicating that, as research and development of CRISPR-related technologies deepened, researchers became increasingly interested in delivery of CRISPR components through EVs. The same trend can be seen in the relative interest in the field. Figure 2B demonstrates that, during the past 7 years, the major part of such publications was produced by more than ten countries (or regions), with Mainland China having the most publications, followed by the USA, Germany, England, and Japan (Fig. 2C). Figure 2D showed that the annual production of articles from the top 10 countries/regions increased from 2 in 2015 to 55 in 2022. Mainland China started to formally publish articles focused on CRISPR related to EVs in 2015, which is earlier than four countries (or regions) in the top 10. However, the growth rate of its publications was faster than for most countries (or regions), except the USA and Germany. Overall, research on CRISPR related to EVs has increased in popularity among researchers in different regions and has reached a rapid developmental phase. The curve of the publication trend is shown in Fig. 2E, indicating that the number of publications in this field increased consistently from 2 in 2015 to nearly 300 by 2050 (*R*^2^ = 0.9220).

### Assessment of publications from different countries and regions

**Fig. 3:**
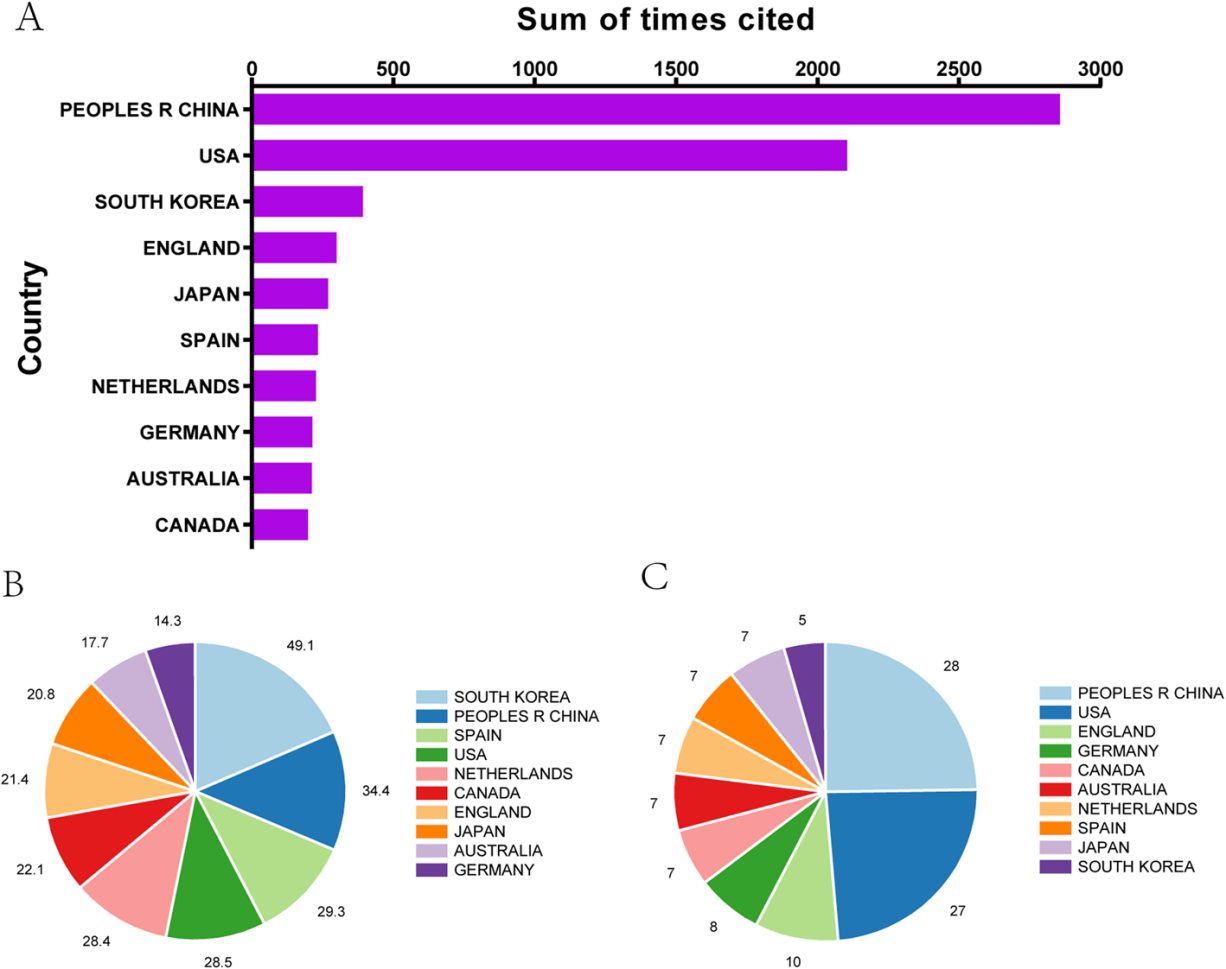
A summary of the citation frequency of related publications worldwide to assess the quality of the publications. **A** The total citation frequency of each country in the top 10. **B** The average number of citations of published articles from different countries. **C** The enumeration and statistics of the highest *H*-index of the countries

Figure 3A shows that publications from the People’s Republic of China have the highest total number of citations (2857), followed by the USA with a total frequency of 2105, South Korea ranked third with 395, England with 299, and Japan with 270. Additionally, papers from South Korea had the highest average citation frequency (49.1). with the People’s Republic of China (34.4), Spain (29.3), and the USA (28.5) ranked second, third, and fourth, respectively (Fig. 3B). The statistics presented in Fig. 3C also indicate that the related publications from the People’s Republic China had the highest *H*-index (28), followed by the USA (27), England (10), anbd Germany (8). Although South Korea had the highest average citation frequency, it had the lowest *H*-index among the top 10 countries (5).

### Bibliometric analysis of global leading authors, countries, and institutions

Analysis of authors can reveal who is representative of a field and acts as the core force. According to Price’s law, authors who have published more than two papers are core authors in this field. According to the statistics of VOS, there are 141 core authors in this field, accounting for 8.4%, which meets Price’s law and indicate that a relatively stable cooperative group of authors has been formed in this field. Table 1 shows the top 6 in this field according to publications.

**Table 1:** Top 6 authors in the field of CRIPSR technology related to EVs

Rank	Author	Publications	Citations	Average citations
1	Wang Y.	6	268	44.67
2	Zhang H.	6	284	47.33
3	Wang J.	5	102	20.4
4	Xu L.M.	5	217	43.4
5	Zhang Y.	5	236	39.33
6	Zhao X.X.	5	355	71

**Fig. 4:**
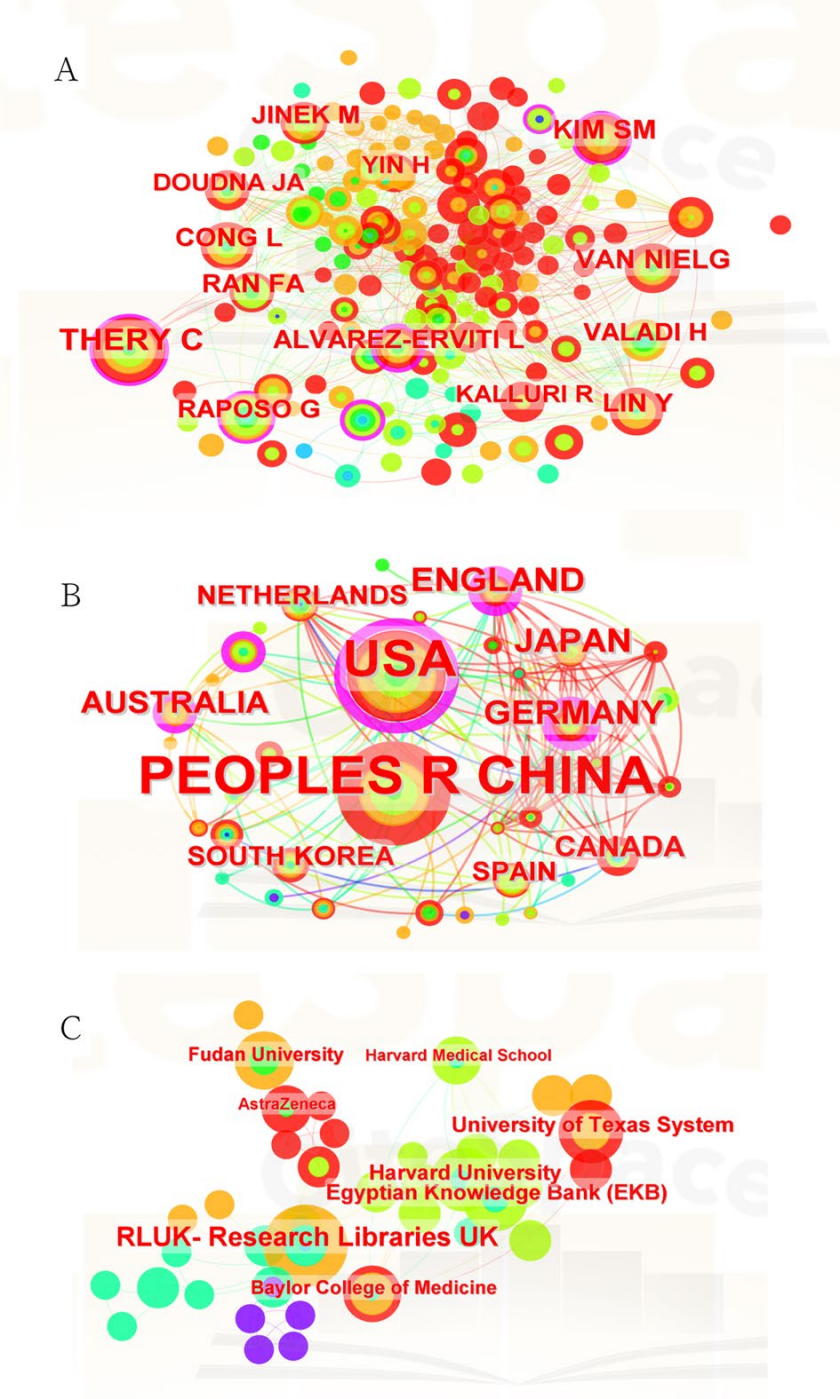
Global leading authors, countries, and institutions. **A** Visualization diagram of authors with highly cited publications. **B** Visualization diagram of countries of highly cited publications. **C** Visualization diagram of institutions producing highly cited publications

Besides the number of publications, the frequency of citations reflects the value of an author's research. We visualize the highly cited authors, countries, and institutions with the corresponding cooperation relationships in Fig. 4.

### Bibliometric analysis of citation bursts of authors, journals, and references

**Fig. 5:**
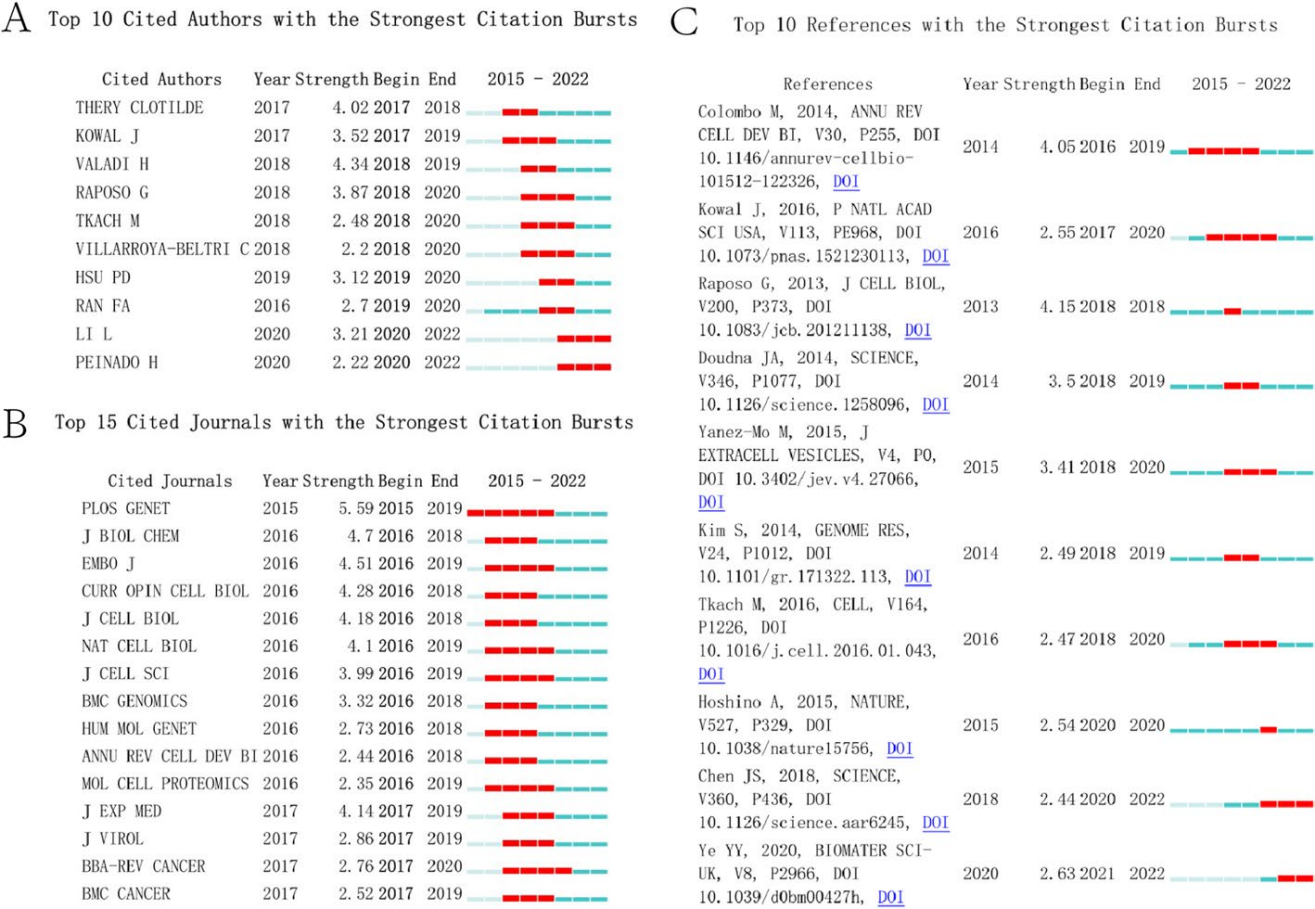
Bibliometric analysis of citation bursts within authors, journals, and references. Red horizontal lines indicate the importance and attention of the authors, journals, and references in the field. A longer red line length indicates greater popularity for authors, journals, and references. **A** Analysis of citation bursts within authors. **B** Analysis of citation bursts within journals. **C** Analysis of citation bursts within references. All items ranked according to “Start year”

The cited author was analyzed and counted according to citation bursts from 2015 to 2022. Citation bursts represent researchers’ attention during a short period, reflecting whether an author’s research is close to the current hotspots. Figure 5A shows that the top 10 authors had strongest citation bursts. The strongest strength is 4.34, produced by Valadi H, followed by Thery Clotilde (4.02), Raposo G. (3.87), Kowal J. (3.52), and Li L. (3.21).

By analyzing the journals, we found that the emergence and development of open-source journals have contributed strongly to the advancement of this research field. There is widespread agreement that research should be freely available, although some scholars currently disagree with open-access journals. Figure 5B shows the top cited journals in this field during the past 7 years. The top cited journal is *PLoS Genetics*, showing the strongest bursts. This academic journal is focused on the field of genetics with a monthly publication cycle. The publication scope of this journal mainly covers genetics. The *Journal of Biological Chemistry* aims to elucidate the molecular and cellular foundations of biological processes through high-quality science, therefore focusing on papers with novel and important insights into mechanisms, rather than in a specific thematic area, making the *JBC* indeed a melting pot for interdisciplinary scientists. Moreover, *EMBO Journal* has published high-quality research with broad interest and impact on molecular and cellular biology, with a focus on physiological correlations and molecular mechanisms. *Current Opinion in Cell Biology* prefers to provide authoritative, comprehensive, and systematic reviews. Highly cited journals with strong bursts mainly involve fields such as genetics, biochemistry, molecular biology, immunology, etc.

The cited publications were further analyzed by first analyzing the top 10 cited papers in the field during 2015–2022 using VOSviewer (Fig. 5C). The first article with citation bursts was written by Colombo, attracting great interest after its publication. This article had a total strength of 4.05 and began to burst from 2016 to 2019. This review comprehensively summarizes the biogenesis, secretion, and subsequent fate of exosomes and other secreted extracellular vesicles [28]. Raposo showed a highest burst strength of 4.15 in 2018 [29]. This review describes the characterization of EVs and currently proposed mechanisms for their formation, targeting, and function. From 2015 to 2022, Kowal showed bursts for the longest period with a total strength of 2.55 [30]. This paper demonstrated the presence of exosomal and nonexosomal subpopulations within small EVs and proposes their differential separation by immuno-isolation using either CD63, CD81, or CD9, thus providing guidelines to define subtypes of EVs for future functional studies.

### Bibliometric analysis of cooperation among authors, countries, and institutions

**Fig. 6:**
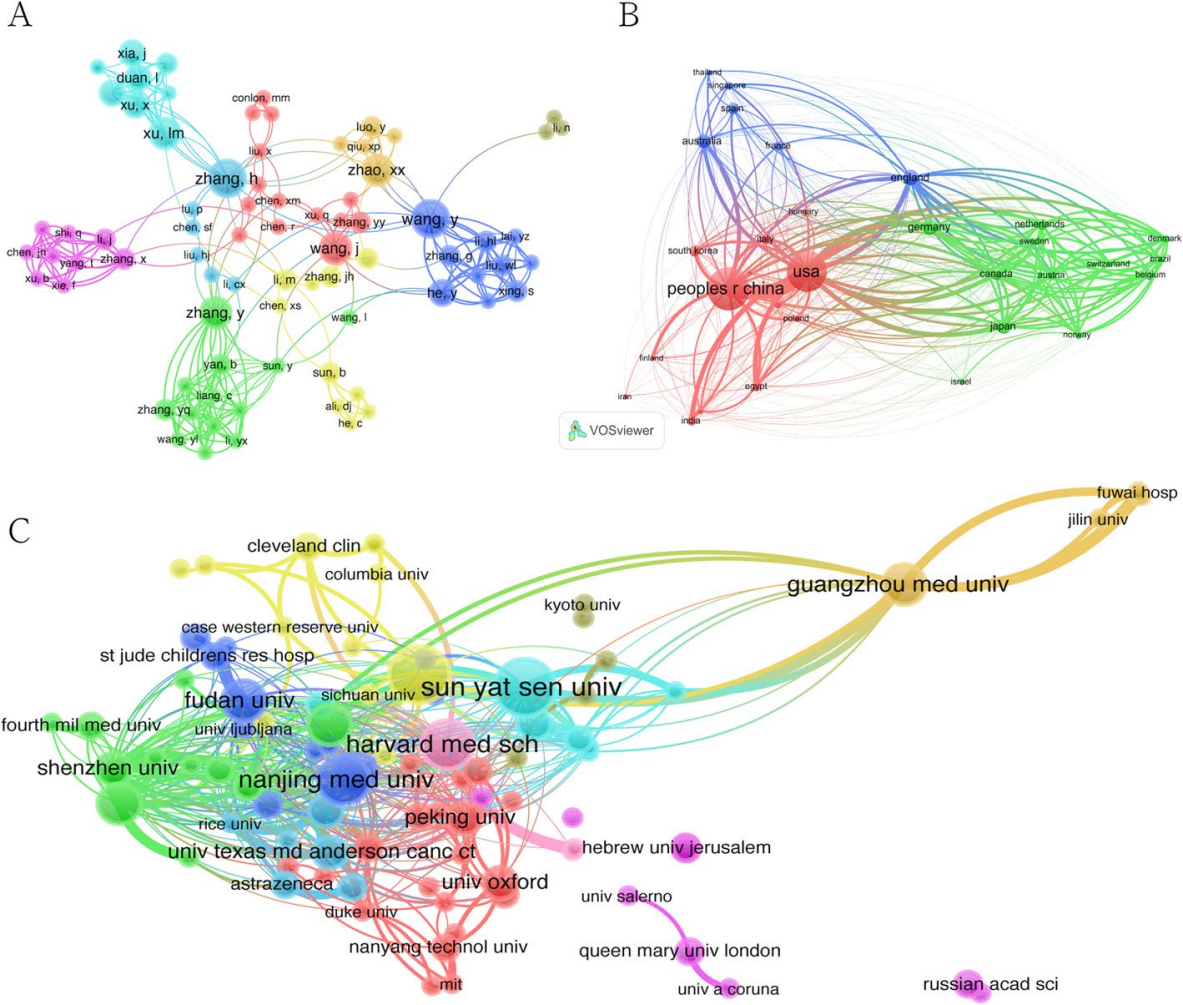
Cooperation map among authorship, countries, and institutions. The size of each node represents the number of publications. The line between nodes represents the cooperative relationship, while the width of the lines indicates the degree of cooperation. Different colors represent different clusters. Nodes of the same color represent authors, countries, and institutions showing closer and more frequent cooperation. **A** Network visualization diagram of co-cited authors of the publications. **B** Network visualization diagram of cooperating countries among the publications. **C** Network visualization diagram of cooperating institutions among the publications

Co-citation means that an article is co-cited by different authors, or an author is cited multiple times. Co-citation can reflect the research direction and cross-development in academia. We counted all co-authored papers and thoroughly assessed the thematic relevance of each entry. We used VOSviewer to analyze all 141 authors who published over two papers. Figure 6A shows that the top 5 authors (where numbers in parentheses represent the total link strengths, as below): Wang [30], Zhang [27], He [25], Xu [24], and Duan [23]. Besides, 31 countries were included to analyze according to the same standard. The corresponding data are depicted and visualized in Fig. 6B. The top 5 countries were found to be the USA (66), England (34), Germany (31), the People’s Republic of China (23), and Canada (20). The top 5 institutions were (Fig. 6C): Guangzhou Med. Univ. (15), Zhengzhou Univ. (14), Chinese Univ. Hong Kong (11), Harvard Med. Sch. (11), and Shenzhen Univ. (10).

In summary, Fig. 6 reveals strong scientific collaboration among authors, institutions, and countries through rich links and nodes. The author cooperation map shows 78 nodes (Fig. 6A). Zhang H., Wang Y., Wang J., Zhang Y., and Zhao X.X. lead in terms of number of publications. The many scientific collaborations between researchers are represented by the dense linkages observed. Clustering effects were identified among the authors and demonstrated with colors (e.g., Wang Y., Zhang G.) and various nodes forming one cluster (Zhang Y.Y., Wang J.) as well as some nodes clustering into another one (Xu L.M., Xu X, Duan L.) and some nodes clustering into one cluster. Figure 6B reveals that the distribution of publications in the field of CRISPR-related EVs is not balanced among different countries. China and the USA account for many publications. They were also the closest research partners, and cooperate closely with many countries, including Australia, South Korea, England, Japan, Germany, etc. Excluding the influence of China and the USA, England, Japan, Australia, Spain, Switzerland, Germany, and other countries also cooperated with each other, but most of them not as closely as with China and the USA. These results confirm that the USA and China have a certain major position. Figure 6C indicates the general cooperation between institutions via nodes and connecting lines. The core institutions in this field include Harvard Medical School, Sun-Yat-Sen University, Nanjing Medical University, Fudan University, etc. Compared with Fig. 6A, the clusters among institutions are not obvious, albeit indicating an extensive, diverse, and abundant cooperation among institutions.

Interestingly, nodes of larger size may not have stronger connecting lines. This condition is particularly evident regarding the countries and institutions. This indicates that the research and paper output in this field is currently restricted by country or geographically. There is a clear internal tendency for researchers to favor working with nationals when collaborating. The core institutions that publish papers are not necessarily those that cooperate more closely with other institutions, which implies “clustering” in current research in this field.

### Bibliometric analysis of co-cited journals and references

**Fig. 7:**
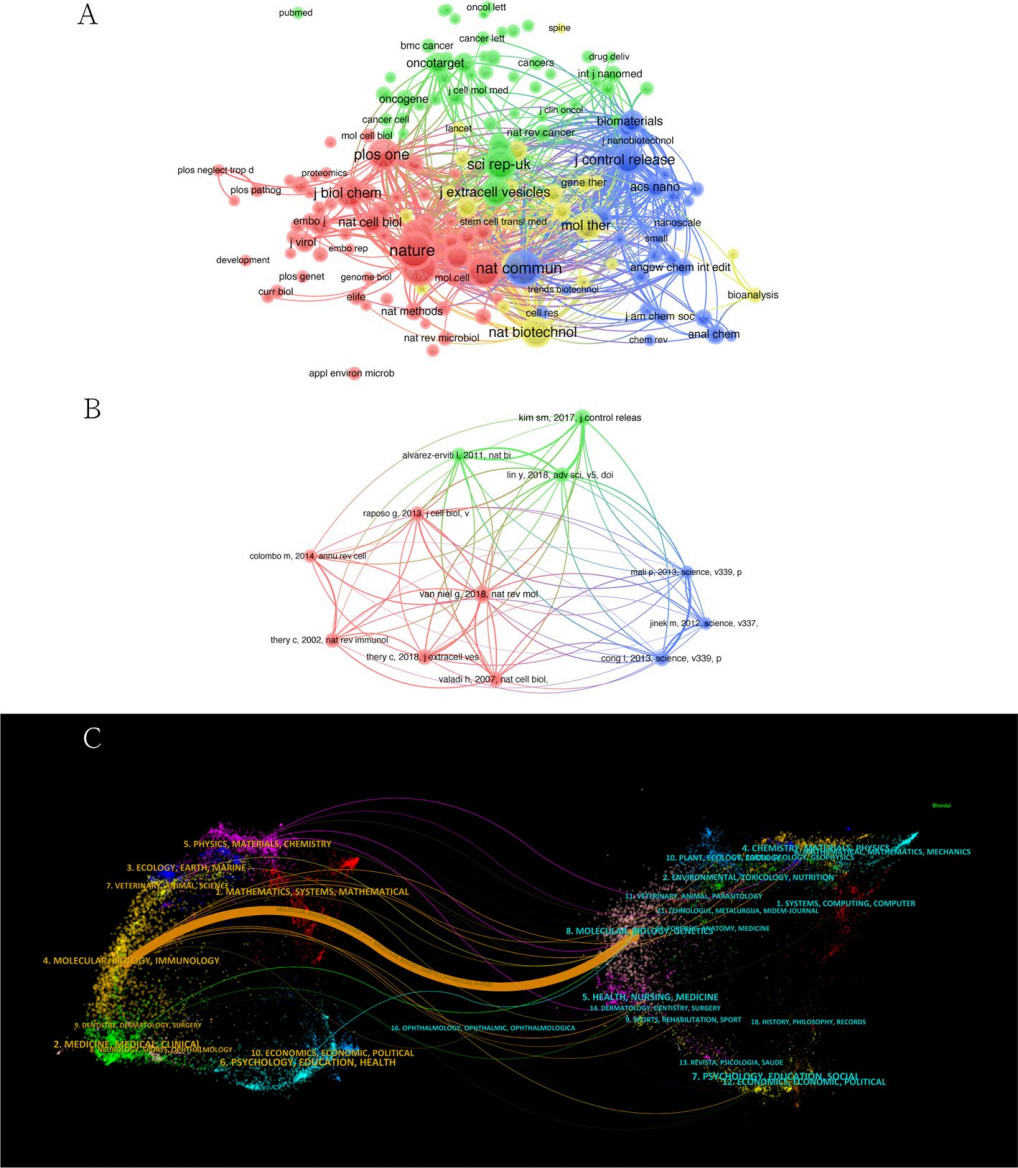
**A** Network visualization diagram of cooperating journals among the publications. **B** Network visualization diagram of co-cited publications. **C** Dual map overlay of journals related to CRISPR associated with EVs

The co-citation network among journals can be seen to consist of roughly four clusters, shown in four colors in Fig. 7A. The top 3 cited journals are *Nature* (citations 419), *Proc. Natl Acad. Sci. USA* (citations 384), *Nat. Commun.* (citations 378). All of these are excellent journals in the JCR1 category.

Among these four clusters, except for the comprehensive journals, the red and blue clusters are mainly related to chemistry and materials, focusing on applications of different types of materials in various aspects. These journals are mainly cited to review and analyze existing studies in the field of materials science and provide previous theoretical and technical support for the development of new materials. The yellow and green journals are mainly in the field of medicine and translational medicine, focusing on specific applications of CRIPSR technology and EVs in the medical field or physiopathological mechanisms behind their therapeutic effects. Citation of publications in this field helps to explore CRISPR-related EVs to broaden understanding of this research area and provide theoretical guidance for the eventual translation of biomaterials and medical engineering.

The co-citation map reveals a co-citation network of highly co-cited literature. Figure 7B shows that this network can roughly be divided into three major clusters, shown in corresponding colors. The blue cluster represents literature in the field of CRISPR/Cas technology [31–33], while the green cluster is mainly related to the application of EVs in delivery systems [34–36]. The publications in the red cluster are mainly literature reviews on the characteristics of EVs [28, 29, 37–40]. Figure 7C shows a predominate citation path in orange, revealing that papers published in molecular/biology/genetics tend to cite papers from this same field.

### Bibliometric analysis of keywords, active topics, and orientation in the future

**Fig. 8:**
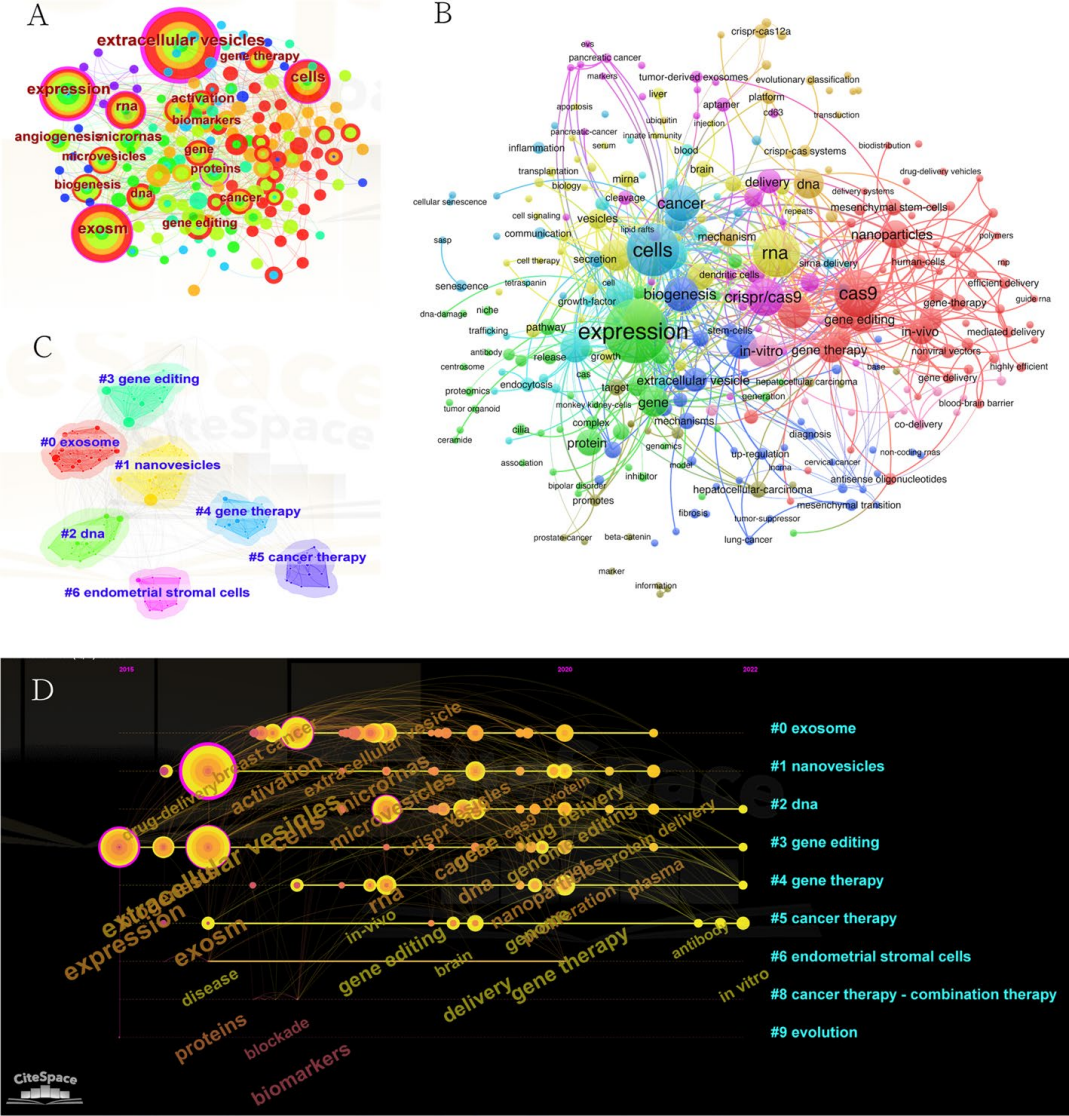
Bibliometric analysis of active topics, keywords, and orientation in the future. **A** Top keywords with the highest citation frequency based on CiteSpace. **B** Co-occurrence analysis of keywords. **C** Clustering analysis of the keyword network based on CiteSpace. **D** Timeline diagram of keywords with corresponding changes of cluster

The objective of keywords and co-occurrence analyses is to identify potential directions and hotspots of research. Such statistics can be used to monitor developments in scientific research. We screened 317 keywords that occurred ≥ 2 times in titles/abstracts within the 219 retrieved papers and visualize them in Fig. 8A. Hotspots in the area are represented by the frequency of these keywords. The keywords are shown in larger fonts and with larger nodes in the map, the more frequently they occurred. To provide a clearer picture of the specific keywords, high-frequency keywords with frequency above 15 are also presented in Table 2. According to Fig. 8A and Table 2, high-frequency keywords such as expression, cells, and Cas9 are representative terms in this field.

**Table 2:** High-frequency keywords in the field of CRIPSR technology related to EVs

Keyword	Frequency	Total link strength
Expression	36	210
Cells	28	158
Cas9	24	154
RNA	24	158
CRISPR/Cas9	18	126
Cancer	17	112
Exosome	17	112
Biogenesis	16	94
CRISPR–Cas9	15	101

The co-occurrence analysis and clusters among keywords demonstrate the internal relationship among keywords. The keyword co-occurrence network view of the total 317 keywords is visualized in Fig. 8B. Larger nodes indicate more frequent appearance and greater representativeness of the domain hotspot; connecting lines represent the association strength, where a thicker line indicates that two keywords appear together in the same publication more often; the node color represents different clusters, i.e., research topics. The keywords identified were classified into seven clusters (Fig. 8C): cluster 0: exosome (red); cluster 1: nanovesicles (yellow); cluster 2: DNA (light green); cluster 3: gene editing (dark green); cluster 4: gene therapy (blue); cluster 5: cancer therapy (purple); and cluster 6: endometrial stromal cells (pink). The clusters indicate the most prominent topics in the field so far. In the “exosome” cluster, the primary keyword was “exosm”. For the “nanovesicles” cluster, the frequently used keyword was “drug-delivery”. For the “DNA” cluster, the main keyword was “microvesicles”. For the “gene editing” cluster, the dominantly used keyword was “extracellular vesicles”. For the “gene therapy” cluster, the frequently used keyword was “biogenesis”. In the “cancer therapy” cluster, the primary used keyword was “breast cancer”. In the “endometrial stromal cells”, the main used keyword was “disease”.

The clustering analysis of the keywords in terms of period allows to derive the baseline status of the research topics within the research field. To clearly identify the temporal patterns of inflection points and frontiers of disciplinary development, the keyword co-occurrence mapping can be arranged in a time series, thus revealing the distribution of research hotspots in each period. In this study, we selected the nodes as “Keywords”, set the “Slice Length” to 1, and set the “Selection Criteria” to “Top 50 per slice” in CiteSpace software, which meant we extracted the data of each time slice, ranking 50 to generate the keyword co-occurrence timeline (Fig. 8D). As seen in Fig. 7D, worldwide research on EVs based on CRISPR-related technologies started to proliferate in 2015 and evolved over a 7-year period. Combined with the qualitative analysis, this can be divided into two phases: the first phase is from 2015 to 2020, and as shown by the keyword clustering, most of the research at that time focused on diseases, disease treatment targets, and exploring the nature of EVs and CRISPR themselves. Since the secretion of EVs must be stimulated by signaling pathways and CRISPR is essentially a tool for gene editing, many keywords related to genetic laws, gene regulation, and targeting sites can be seen. This phase of research topics is relatively more focused. The second phase is from 2020 to 2022, when keywords begin to appear decentralized, with expanding research themes and a continuous increase in research hotspots. These include the selection of specific delivery options, in-depth exploration of CRISPR and vesicle-related technologies, well-defined gene therapies, and the study and exploration of a range of biomaterials, mainly nanomaterials. This indicates that scholars from different backgrounds and directions are converging here, while the research is moving from mechanism exploration and analysis and technology validation to the field of translational medicine, step by step.

## Discussion

### Trends in global publications

CRISPR-related technologies have been widely explored and used to achieve better effects within clinical and basic research [41]. However, numerous challenges concerning efficacy and safety remain to be addressed for their full clinical application, such as the fitness of edited cells, the editing efficiency, delivery methods, and potential off-target effects [41–45]. Therefore, EVs, including macrovesicles, exosomes, ectosomes, microparticles, apoptotic bodies, and oncosomes, have been regarded as replacement tools for conventional delivery strategies because of their fewer side effects, ability to protect therapeutic agents and cross biological barriers, lower immunogenicity, easier preservation methods, and fewer ethical issues [46]. In recent years, numerous researchers have proposed meaningful strategies in the field of CRISPR-related technologies associated with EVs. Liu et al. [47] described the trends and status in CRISPR-related cancer therapy field from 2013 to 2022, and pointed out research directions of cancer-related gene editing, mechanisms, and oncogenic molecules. A bibliometric study on mesenchymal stem cell-derived EVs was conducted by Zhang et al. to explore the mechanisms, therapeutic effects, and an overview of the growth trend [48]. The senescence-related and multidisciplinary integration topics might be hotspots according to bibliometric analysis. However, visualized analysis of worldwide trends of EV-based delivery systems of CRISPR-related components has not been studied yet. Thus, we conducted this study and found persistently increasing trends and RRIs of such publications. In particular, China has contributed the most papers, followed by the USA, Germany, England, and Japan. On the basis of increasing findings of intercellular communication in EVs under various conditions in vitro, studies on EVs used in different disease models have been conducting frequently [49, 50]. All of these indicated that EV-based delivery systems for CRISPR-related components will be a research direction with brilliant prospects.

### Proportion of literature published according to different types of EVs

EVs can be secreted by all cells and are membranous, nonreplicating, nanoscale particles capable of acting as signaling molecules with communication roles between paracrine and endocrine cells [51, 52]. Despite their significant overlap and heterogeneity, the main basis for grouping EVs is their size and biological origin. For example, vesicles released outside the cell with the cytoplasmic membrane are called exosomes, while vesicles produced by budding through the cytoplasmic membrane are called extracellular bodies or microvesicles [53–55]. As applied in previous strategies, we focused our search on extracellular vesicles (138), exosomes (139), ectosomes (0), apoptotic bodies (9), microvesicles (22), oncosomes (1), and microparticles (6), where the number in parentheses represents the number of documents found in the search for that keyword alone. Owing to the intersection and overlap among the keywords, there will be many duplicate publications when searched individually, thus their sum will exceed the total number obtained through the total search formula. We found that most of the literature is currently focused on the two main categories of extracellular vesicles and exosomes, both of which will remain as research hotspots in the field for a long time owing to the continuous development of various functionalization methods.

### Quality and status of global publications

We evaluated the academic impact and quality of publications from different countries; Fig. 3 shows the *H*-index and citation times. Although South Korea exhibited the highest average number of citations, China contributed the most publications, *H*-index, and citations. The existence of this phenomenon may be due to the specific assessment system of China, which tends to focus on achievements recently (18). Although great progress has been achieved, the average number of citations demonstrates that Chinese researchers are supposed to improve the quality of publications. Besides, the leading institutes contributed more to this field. Interestingly, nearly all the top institutes are in the top countries, demonstrating the key role played by high-level institutions in promoting national academic rankings. Finally, we analyzed the related journals, showing the result in Fig. 7. Top journals are usually regarded as the most valuable and potential platforms for publishing such papers.

This bibliometric study could provide guidance for focused research fields. Thus, we mapped co-occurrence figures among all the involved authors, institutions, journals, and countries (Figs. 4, 5, 6, 7 , and  8). Specific results are mentioned above. Co-citation analysis was also conducted to determine the impact of publications. These results may assist future researchers to learn about the field quickly and help identify who to learn from.

### Research focuses on CRISPR-related technologies associated with EVs

The popular topics and directions in CRISPR-related technologies associated with EVs were evaluated via keyword co-occurrence analysis. We reviewed the characteristics of related publications from 2015 to 2022, structurally and temporally. The CRISPR-EV field still has hot status, with an obvious increase in the production of publications, extension and relationships of collaborations, and dense and tight citation networks. We predicted potential hotspots through citation bursts and keywords analysis. Keywords such as exosome, nanovesicles, DNA, gene editing, gene therapy, cancer therapy, and endometrial stromal cells may become more popular in the future. We also find that multidisciplinary work has become a new trend, via interpreting the shifts of subject category bursts, especially related to medical engineering and biomaterials. In addition, the visualized timeline of keywords indicates that directions such as drug delivery, extracellular vesicles, expression, and exosomes are conventional terms that are consistently present in CRISPR-EV research, while protein delivery, nanoparticles, and antibody have become emerging research modules.

### Exploration of emerging topics

In addition to popular terms such as extracellular vesicle, cell, gene expression, exosome, etc., which are widely followed and searched for, many of the emerging research terms can be identified from Fig. 8D, especially the emergence of timeline plots predicting trends in research development over time. For instance, regarding drug delivery, it has been found that the conventional viral delivery system itself has some insurmountable limitations. Waqas et al. [56] produced large quantities of erythrocyte-derived EVs for RNA drugs such as Cas9 mRNA. In human cells and xenograft mouse models, this class of RNA drugs showed robust CRISPR-Cas9 genome editing capabilities after delivery without cytotoxicity. Yao et al. also proposed a strategy to deliver drugs using RNP-enriched EVs as delivery systems with efficient genome editing and transient expression capabilities [46]. The application of EVs in delivery of CRISPR-related components presents a novel concept with meaningful achievements from vivo/vitro research, but limited data are available; data collected from long-term follow-up clinical studies is required to ensure efficacy [57]. Regarding nanoparticles, although engineering of EVs for RNA delivery has been extensively studied, we do not known the precise difference in efficiency of RNA delivery by EVs themselves compared with currently used synthetic RNA delivery vectors. Current studies usually focus on comparison between EVs and nanoparticles when used as a delivery system [58]. Co-delivery systems targeting nanoparticles combined with extracellular vesicles may be a key direction for future research. Regarding proteins, these are not only the product of genetic engineering, but also an important tool to be able to modify extracellular vesicles. Strohmeier [59] provides CRISPR/Cas9-modified fluorescent proteins to label markers of extracellular vesicles, which enhances the function of extracellular vesicles and contributes to the final tracing. Whitley [60] found a method for modifying EVs using protein myristoylation, which facilitates the encapsulation of CRIPSR products through preferential encapsulation of myristoylated proteins to generate substrates, providing a novel approach for the encapsulation of CRISPR/Cas9 proteins and sgRNAs into EVs. Regarding plasma/antibodies, microRNAs in EVs play an important role in tumorigenesis and progression. Quantitative determination of these microRNAs is essential for cancer diagnosis and longitudinal monitoring. CRISPR modification technology can encapsulate CRISPR/Cas13a components in liposomes and deliver them to EVs, opening up the possibility of multiple single-EV analyses of protein and RNA markers in plasma though sensing function [61]. Regarding gene therapy, the CRISPR/Cas9 system has attracted more attention for use in potential gene therapies for precision medicine, especially for genetic diseases [62]. Valence-controlled tetrahedral DNA nanostructures (TDNs) containing DNA aptamers were designed and loaded onto the EV surface by cholesterol anchoring for cell-specific targeting by Zhuang et al. [63]. This study proved the feasibility of delivering components for cell-selective gene edition. Other research conducted by Majeau indicated that serum EVs could be used to deliver CRISPR-CAS9 ribonucleoproteins to modify the dystrophin gene [64]. Current studies thus demonstrate that EV-based CRISPR technology has a brilliant future in the field of gene therapy.

## Further directions

Bibliometric studies can summarize the dynamic development trends in a research field comprehensively. In this study, we review the publications in the last 7 years and summarize the corresponding burst keywords. This study can provide future researchers with comprehensive understanding of the development process of this field through a systematic review of the relevant publications. Besides, the core institutions and authors in the field were identified to assist researchers to clarify the target audience for learning and to seek collaborations within their scope of competence. Additionally, core journals in the field were listed, and researchers can obtain the latest progress through long-term and continuous attention to these journals. Finally, through the quantitative analysis of the keywords we performed, researchers can identify potential future research directions.

In summary, further research could be conducted in the following aspects: (1) Data mining and exploration through Big Data platforms on CRISPR-EV research. Data mining can be an important step for revealing implicit, unknown, and useful information from various databases, thus helping decision-makers tailor their evaluations to reduce potential risks. Finding potential targets for disease treatment through a variety of Big Data platforms, such as bioinformatics and public databases, and searching for editing sites for diseases and genes by CRISPR technology is already a more mature idea and will continue to be one of the trends for future development in this field. (2) EV-related biomaterial design. The development of biomedical engineering brings rapid development of biomaterials. EVs under modification by multiple biomaterials will make them better for use as nanoscale structures for delivery of CRISPR-related products and make it possible to realize targeted and individualized therapies by specifically designing them for corresponding diseases and potential sites. (3) Multicomponent and multidrug synergy. By increasing research on extracellular vesicles and CRISPR technology, multiproduct or multisite editing synergy may become one of the directions for future research. Challenges in this area may arise more from the choice of sites, how well CRISPR is encapsulated within EVs, and how its own biological capacity is effectively preserved. (4) More rigorous clinical studies. Cas9-based target screening provides an advanced method for better understanding of many diseases. However, owing to the off-target effect, administration challenges, limitations, and immunogenicity, further clinical application of CRISPR is hindered [65]. Although EVs have potential for delivering CRPSIR/Cas products, owing to source issues, many EV biomacromolecules have the potential to affect cellular physiological functions, such as tumor-derived EVs, which may lead to tumor development and metastasis [66]. The consistency of the quality and production of EVs play an indispensable role in the clinical application of EVs, which limits their clinical applications [67]. These potential dangers lead to more careful ethical considerations when approving clinical trials related to EVs. While extensive preclinical studies have been conducted, we need more clinical data to reveal the eventual true efficacy and validate safety in the clinic, to better promote its development in the field of translational medicine.

## Limitations of this study

Although we have ensured that this study offers an overview and guidance for research in CRISPR-related technologies associated with EVs, some limitations remain. Firstly, it is hard to explain details about EV-related delivery strategies, for example, the origin and types of EVs and their relationships, and the influence on the choice of CRISPR technique. Secondly, some publications may have been omitted because of bias in terms of database or language, such as publications from Cochrane, Embase, PubMed, or Scopus, and non-English-language publications. In addition, we only included articles and reviews, so the most advanced results presented in the form of posters or conference reports may have been omitted. Thirdly, the newest, high-quality papers may not be included because of their weak citation, resulting in a gap between the bibliometric analysis and the real world. We thus suggest that researchers pay attention to the newest publications, especially papers in languages other than English.

## Conclusions

We conducted this study to demonstrate the status and global trends in CRISPR-related technologies associated with EVs from 2015 to 2022. China contributes the most publications, showing the highest *H*-index and citations in this field. In addition, The International Journal of Molecular Sciences [10] published most papers related to this topic. Research on EV-related CRISPR technologies will receive more global attention, and exosome, nanovesicles, DNA, gene editing, gene therapy, cancer therapy, and endometrial stromal cells will be hotspots in the future. Finally, the keyword burst detection indicated that drug delivery, nanoparticles, protein, plasma/antibody, and gene therapy might be emerging research hotspots.

## Data Availability

The data that support the findings of this study are available on request from the corresponding author (Xintao Zhang), upon reasonable request.

## Abbreviations

GE: Genome engineering
CRISPR/Cas: Clustered regularly interspaced short palindromic repeat (CRISPR)/CRISPR-associated (Cas)
sgRNA: Single-guide RNA
WoSCC: Web of Science Core Collection
EVs: Extracellular vesicles
RRI: Relative research interest
TDNs: Tetrahedral DNA nanostructures
